# Addition to On the Move-Sensitive Fluorescent Aptassay on Board Catalytic Micromotors for the Determination of Interleukin-6 in Ultra-Low Serum Volumes for Neonatal Sepsis Diagnostics

**DOI:** 10.1021/acssensors.2c02731

**Published:** 2023-01-09

**Authors:** José Gordón, Luis Arruza, María Dolores Ibáñez, María Moreno-Guzmán, Miguel Ángel López, Alberto Escarpa

This addition is the sequence of interleukin-6 (IL-6) aptamer: 5′-GTT GCT CGT ATT TAG GGC CGA GGT ACG AGT GTC TTT GGG ATC TGC ATT CTC CTG CGT GAC ACC AGT CTT CAT CCG C-3′

